# The Views of Medical Students on Elder Discrimination

**Published:** 2012-05-30

**Authors:** O Ogenler, G Yapici, B Tasdelen, T Akca

**Affiliations:** 1 Department of History of Medicine and Ethics, Mersin University Faculty of Medicine, Mersin, Turkey; 2 Department of Public Health, Mersin University Faculty of Medicine, Mersin, Turkey; 3 Department of Biostatistics, Mersin University Faculty of Medicine, Mersin, Turkey

**Keywords:** View, Medical student, Elder, Discrimination

Dear Editor,

The age of 65 and older grows rapidly in the world, which until recently was deemed to be young. Changes in the social structure increase the importance of nursing elderly patients in the area of health services.[1][2] The most important problem is the fact that health services can not be allocated fairly as it is implemented worldwide. While fairness in nursing of elderly patients takes shape with the equal usage of health services, it diminishes in line with the health personnel's tendency to discriminate elders.[1][3]

Elder discrimination is defined as discrimination aimed at the elderly persons. Only behavior patterns depending on chronological age develop in health personnel when elder discrimination exists and a tendency to treat the acute diseases of the young persons rather than dealing with the elders' chronic diseases arises.[3][4][5][6][7]

In our research, it has been aimed to determine the attitudes of the medical students concerning age discrimination and the factors affecting these attitudes. This cross-sectional study was conducted on 63 sixth grade students studying in Faculty of Medicine during the 2009-2010 academic year. The research protocol was approved by the Faculty Executive Committee.

The survey was composed of “Elder Discrimination Attitude Scale (EDAS)”. The scale consisted of 23 statements that incorporated both positive and negative judgments concerning elders, senescence or aging. The total point that a participant could achieve with EDAS ranged between the values 23 and 115. Scoring a higher total point indicates the fewness of negative attitudes in respect of elder discrimination.

Descriptive statistics (mean, standart deviation, median, and range) were used for summarize the data. The Chi-Square test was used to compare the categorical variables. Comparisons of continuous variables were made using Student’s t-test and Mann-Whitney U test. p<0.05 was adopted as a statistically significant difference.

Average age of the 63 students (as 21 females and 42 males) included in the research was 24.08±1.46 years (range 22-29 years). The median EDAS score was 87. There was no statistically significant relationship between the students' EDAS scores and their sex, place of birth, places of residence, types of family, whether they live with elders or not, and the places where their grandparents lived. Total EDAS points of the students who did not want to live with their own parents after getting married were found out to be higher than that of those who wanted to live with their parents after getting married (Table 1).

**Table 1:** Distribution with regard to elder discrimination attitude scale

		**No.**	**Median**	**Min- Max**	***P* value**
**Sex**	Male	42	86	51-105	0.439
	Female	21	88	77-106	
Birth place	Province	27	85	51-100	0.077
	Town	30	87.5	69-106	
	Village	6	93.5	81-101	
Residence	Alone/Student hostel	11	84	70-92	0.317
	Living with parents or relatives	23	87	51-103	
	Living with friends	29	89	69-106	
Types of family	Nuclear family	57	87	51-106	0.860
	Broad family	6	86	81-101	
Lived with elders	Yes	26	85	77-101	0.105
	No	37	89	51-106	
Residence of grandparents	Their family	10	84	79-101	0.662
	Uncle	18	88.5	74-102	
	Alone	20	86,5	51-106	
	Do not live	13	89	77-103	
Want to live with their parents	Yes	33	85	51-103	0.024
	No	29	90	70-106	

While the EDAS total point of the students who, in a period of time, lived with elders and who intended to live with their parents in the future was 83.9±4.1 and the EDAS total point of the students who did not intend to live with their parents was 88.4±8.1 (p=0.102). The EDAS total points of those who had never lived with elders in the same house and stated that they could live with their parents in the future was 85.7±12.7. The same was noticed for those who had never lived with elders and stated that they did not intend to live with their parents in the future which was 93.3±9.9 (p=0.086).

The students who had participated in our research had a positive attitude, resulted from the traditional value of respecting the elders as a constant expectation. There are researchs in the literature showing that students receiving medical education have negative attitudes towards the elders.[8] In our research, female students had higher EDAS points than the male students. Our research showed that the female students had accepted the traditional ones.[2][7]

The students' circumstances of living with elder people for all their lives, their grandparents living together with their families, and their desire to live with their own parents in their future lives brought in a more negative attitude towards the elders. Although most of the students who wished to live with their parents in the future had stated that they did so due to their love towards their parents, in our study their total EDAS points turned out to be lower than that of those who stated that they did not want to live with their parents in the future. An idea of special responsibility between the relatives was contradictory with the principle of ethical impartiality.[5][6] Age discrimination in terms of doctor-patient relationship is important. For this reason, althougt the students have generally positive attitudes, negative attitudes of them need to be corrected. In conclusion, increasing the awareness of health personnel and especially doctor candidates, providing knowledge on elder discrimination during all phases of medical education, development of clinical and ethical rules by professional organizations, application of enforcements through laws by the governments and ensuring the participation of those who received medical services can remove the discrimination made against the elders.
